# Prevalence and diversity of gastrointestinal helminths in free-ranging Asian house shrew (*Suncus murinus*) in Bangladesh

**DOI:** 10.14202/vetworld.2018.549-556

**Published:** 2018-04-30

**Authors:** Mizanur Rahman, Shariful Islam, Md. Masuduzzaman, Mahabub Alam, Mohammad Nizam Uddin Chawdhury, Jinnat Ferdous, Md. Nurul Islam, Mohammad Mahmudul Hassan, Mohammad Alamgir Hossain, Ariful Islam

**Affiliations:** 1 Department of Pathology and Parasitology, Faculty of Veterinary Medicine, Chittagong Veterinary and Animal Sciences University, Chittagong-4225, Bangladesh; 2 EcoHealth Alliance, New York, USA; 3 Institute of Epidemiology, Disease Control and Research (IEDCR), Mohakhali, Dhaka-1212, Bangladesh; 4 Department of Animal Science and Nutrition, Faculty of Veterinary Medicine, Chittagong Veterinary and Animal Sciences University, Chittagong-4225, Bangladesh; 5 Bangabandhu Sheikh Mujib Safari Park, Gazipur-1741, Bangladesh; 6 Department of Physiology, Biochemistry and Pharmacology, Faculty of Veterinary Medicine, Chittagong Veterinary and Animal Sciences University, Khulshi, Chittagong 4225, Bangladesh

**Keywords:** Asian house shrew, Bangladesh, gastrointestinal helminths, prevalence, *Suncus murinus*

## Abstract

**Background and Aim:**

Asian house shrew (*Suncus murinus*), a widely distributed small mammal in the South Asian region, can carry helminths of zoonotic importance. The aim of the study was to know the prevalence and diversity of gastrointestinal (GI) helminths in free-ranging Asian house shrew (*S. murinus*) in Bangladesh.

**Materials and Methods:**

A total of 86 Asian house shrews were captured from forest areas and other habitats of Bangladesh in 2015. Gross examination of the whole GI tract was performed for gross helminth detection, and coproscopy was done for identification of specific eggs or larvae.

**Results:**

The overall prevalence of GI helminth was 77.9% (67/86), with six species including nematodes (3), cestodes (2), and trematodes (1). Of the detected helminths, the dominant parasitic group was from the genus *Hymenolepis* spp.(59%), followed by *Strongyloides* spp.(17%), *Capillaria* spp. (10%), *Physaloptera* spp. (3%), and *Echinostoma* spp.(3%).

**Conclusion:**

The finding shows that the presence of potential zoonotic parasites *(Hymenolepis* spp. and *Capillaria* spp.) in Asian house shrew is ubiquitous in all types of habitat (forest land, cropland and dwelling) in Bangladesh. Therefore, further investigation is crucial to examine their role in the transmission of human helminthiasis.

## Introduction

Shrews, belonging to the order Insectivora and under the family of Soricidae and the genus *Suncus*, are extensively distributed in Asia, Africa, and Europe [1]. Of the 18 currently recognized species of *Suncus*, only two species, namely *Suncus murinus*, the house shrew (Chika or Chucho), and *S. etruscus*, the pygmy shrew (Baman Chika), are native to Bangladesh. The first one is very common and found in the holes and drains of urban and rural areas and forests, whereas the latter one is very rare, and no recent sight has been recorded [2]. Shrews’ feed habit is restricted almost entirely to invertebrate prey. Shrew plays an important role as reservoirs and hosts of many pathogens of animals and humans [3]. They are commensal with humans and are primarily found near human habitation and other synanthropic habitats such as rice fields and grain warehouses [4]. Asian house shrew is the natural reservoir of *Thottapalayam virus* [5], Hantavirus [6], Plague [7], and *Toxoplasma gondii* [8].

The invertebrate diet of shrews predisposes them to infestation by helminth parasites. Helminth parasites in small mammals comprise four major taxonomic groups: The cestodes or tapeworms, the flukes, the nematodes or roundworms, and the acanthocephalans or spiny-headed worms. Almost all of the cestodes, flukes, and acanthocephalans, as well as many of the nematodes, require one or more invertebrate intermediate hosts for the development of their larval stages to complete their life cycles [9]. The vertebrate’s definitive host becomes infected either as a result of direct penetration of their tegument by larval stages or by ingestion of infective stages, the latter being the more usual route, especially in the case of helminths of terrestrial vertebrates [10,11]. Thus, the shrew will become infected with helminth parasites through its food and is likely to harbor a more diverse helminth fauna than the other sympatric small mammals. This has been confirmed by previous studies of British small mammals [11,12].

Parasites have a key role in ecosystem, affecting the ecology and evolution of specific interaction, final host population growth, and biodiversity of the community [13,14]. Ecology may have direct pathological effects on host survival and reproduction, whereas indirect effect may be the reduction of the hosts’ body condition [15]. Anthropogenic land use change is leading to novel interactions among ecological factors related to vectors, hosts, and disease [16]. Predicting the influence of land gradient on parasite incidence is important but challenging. Because, very little is known about many wildlife diseases, especially in context of Bangladesh where no baseline study was done on shrews.

In different studies, factors such as habitat selection, elevation distributions, diet, nest type, and participation in colonies or mixed-species were found to influence parasitic infestation rates [17] in host or vector community [18]. Many studies have been conducted on evaluating the prevalence of parasites among wild rats throughout the world [4,19]. Although there have been several reports of helminth infestations in shrews and rodents from other parts of the world [4,20], but studies from Bangladesh are very limited. So far known, no epidemiological and molecular data exist regarding the helminth infestations and diversity among shrews in Bangladesh.

There are several diagnostic techniques for parasite species identification such as qualitative, quantitative, and culture. Among different diagnostic methods, qualitative techniques are more convenient, easy to perform, and economic. Every technique has several limitations. It is difficult to differentiate different species of parasites using single diagnostic technique. To avoid such types of limitations, combined recovery of eggs from fecal samples using flotation and sedimentation is recommended [18] along with direct smear. Collection of gross parasites by necropsy and identification of parasites provide more accuracy of species identifications.

The close association between insectivores, humans and their livestock along with their exposure to blood-sucking arthropods, beetles, cockroaches, and other invertebrates, extends the scope for transmission of helminths. The parasitic infestations that shrew harbor and convey to human or animal populations have not been thoroughly investigated, especially in over-populated countries like Bangladesh. As the density of human population continues to increase exponentially, speeding the reduction and fragmentation of animal habitats, greater human-animal contact is inevitable, and even higher rates of pathogen transmission are likely. Thus, interest has grown in collecting baseline data on patterns of parasitic infestations in shrew’s populations as an index of their health and to assess and manage disease risks to human.

Therefore, the study was aimed at understanding the diversity and prevalence of helminths fauna of Asian house shrews and the role of environmental factors in facilitating helminth infestation in *S. murinus* in Bangladesh.

## Materials and Methods

### Ethical approval

The Animal Experimentation Ethics Committee of Chittagong Veterinary and Animal Sciences University, Bangladesh (CVASU-AEEC), approved the study protocol and the approval number for the project was CVASU/Dir (R and E) AEEC/2015/07.

### Study period and area

**Figure-1:**
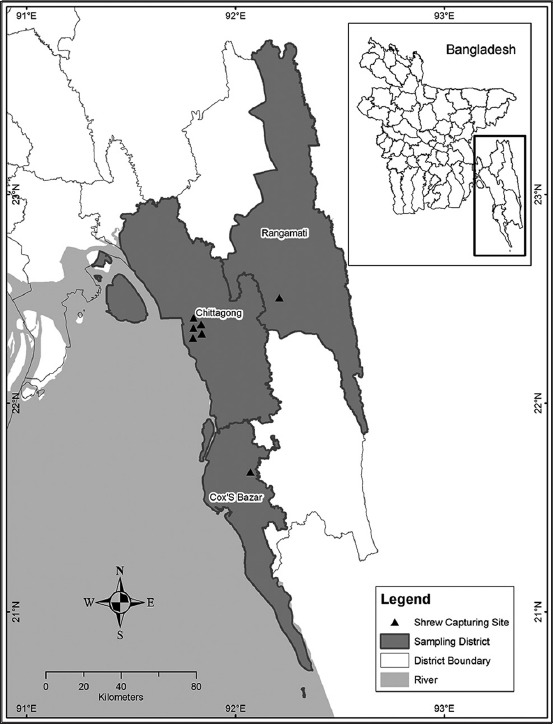
Distribution of shrew sampling sites.

A 6-month-long cross-sectional study was conducted in Chittagong division, Bangladesh, between July and December 2015. Three major sites were selected, with each location representing unique geographical location, and the areas were naturally decorated with mountains, rivers, forests, valley, and sea in southeastern part of Bangladesh. The studied areas are Chittagong Metropolitan area, Kaptai area of Rangamati district and,Chakaria area of Cox’s bazar district of Bangladesh (Figure-1). All the sites were characterized by subtropical climate and high humidity throughout the year, with temperature ranging between 26°C and 32°C and heavy rainfall coincided with the monsoon season (June to October).

### Trapping of shrews

A total of 86 individuals of *S. murinus* (52 males and 34 females) were trapped from the study areas using live traps. Locally made steel wire cage traps (measuring 27 cm × 13 cm × 13 cm) in each sampling location site have proven correct efficiency in a preliminary sampling of medium- and large-sized shrews. All the traps were baited with ghee smearing biscuit and dried fish [21-23]. Every day, 42 traps were set at dusk and checked at the next dawn. The exact locations (latitude and longitude) of the habitats and traps area were recorded using a Global Positioning System device.

### Sample collection

Isoflurane was used for induction of anesthesia followed by the killing of shrews inside a ziplock bag. The shrews were only handled out of the zip bag after been deeply euthanized, as soon as breathing and heart beating has stopped [22,24]. The captured Asian house shrews were identified based on morphometric parameters described by Payne *et al*. [25].

The shrews were dissected to dismantle all the internal organs where the helminths may be found. All the harvested organs were carefully searched for the presence of helminths in the presence of physiological saline. The whole gastrointestinal (GI) tract was preserved in a plastic box, specimen container, or in a plastic bag, filled with 70% alcohol as a fixative agent. A certain amount of feces was collected and stored in formalin for coproscopy. After removal of GI tract from the shrews, the remaining parts were kept in biohazard bags and the bags were properly buried.

### Identification of helminths

The gut sample was spread out in a Petri dish. Few drops of 70% alcohol were poured into the Petri dish to facilitate the dissection. The direct smear was taken from the inner side of the specimens and then the smear was examined under a stereomicroscope. The gross helminths were carefully collected and relaxed in 2% lidocaine, then fixed in 70% alcohol, and stained by Mayer’s Paracarmine [21]. The eggs were counted under the stereomicroscope.

### Staining of nematodes and cestodes

Nematodes were examined using Amman’s lactophenol. For cestodes, parasites were relaxed by placing them in warm saline and then flattened by placing them in between two slides and compressing the slides together using an elastic band. The pair of slides was placed in slightly hot alcohol-formalin-acetic acid solution for 20 min to fix the parasites in a flattened state, allowing the internal structures to be seen more clearly. After flattening, the specimens were transferred through an alcohol series to 70% alcohol before staining in Mayer’s Paracarmine.

### Measuring of parasites

The parasites were transferred from the xylene onto a microscope slide using a brush with three bristles. A couple of drops of Canada balsam, DPX, or XAM were placed on the top of the specimen and a coverslip was gently lowered on the top of it using a mounted needle. Measurements of the various structures of the parasites were made using a micrometry.

### Coproscopy

All fecal samples were examined under a compound light microscope both in wet mounts and flotation technique. Slides were initially examined in low magnification (10x) to trace followed by high magnification (100x) for identification of eggs or parasites [26,27]. Eggs of particular species and gross parasites were identified based on morphological characteristics (shape and shell structure) and size as described by Foreyt [27] and Sloss and Kemp [28].

### Statistical analysis

The raw data were recorded accordingly using MS Excel 2013. The descriptive analysis was performed using STATA 13 (StataCrop, 4905, Lakeway Drive, College station, Texas 77845, USA). Associations among variables such as host origin, sex, age, anthropization, and parasitization were calculated by Chi-square (λ^2^) test or Fisher’s exact test when necessary. p<0.05 was considered statistically significant. The mean worm load was calculated by formula described by Opara and Fagbemi [29]. The mean worm load for each taxa was calculated as number of worm of each taxa from shrews of each land gradient divided by the total number of shrew positive for that parasitic taxa in that land gradient. Relative abundance was calculated as the total number of worm of each taxa found divided by the total number of shrew examined in a specific land gradient. The overall relative abundance was calculated as the total number of worm found in that land gradient shrew divided by the total number of shrews tested of that land gradient.

The helminth diversity was calculated following Shannon index, H = ∑(P1) IlnP1I [30], where P1 is the proportion of the total number of individual in the population that are in species “l.” The map of the study area was made based on the latitude and longitude collected during sampling using ArcGIS software.

## Results

Based on gross parasite samples’ morphometric characteristics and eggs’ morphology, six helminths taxa were detected. Helminth genera included *Hymenolepsis* spp., *Taenia* spp., *Capillaria* spp. (Capillariidae), *Strongyloides* spp. (Strongyloididae), *Physaloptera* spp. (Physalopteridae), and *Echinostoma* spp. In forest areas, *Taenia* spp. and *Physaloptera* spp. were prevalent, whereas in croplands, *Echinostoma* spp. was prevalent, but in human settlement areas (dwelling), all of the taxa were present.

### Overall prevalence

The prevalence, mean worm load, relative abundance, and species diversity of GI parasites were examined in accordance with the land gradient-forests, croplands, and dwelling areas. Of the 86 shrew from different land gradients, 67 (77.91%) were infected with one or more species of GI parasites, where cestode was the most prevalent than others (63%). However, the nematodes and trematodes were found by 31% and 3%, respectively. The cestodes were most prevalent in dwelling area, whereas nematodes and trematodes were more prevalent in croplands and forest areas. The most prevalent parasite was *Hymenolepis* spp. (59%), followed by *Strongyloides* spp. (17%), *Capillaria* spp. (10%), and others (Table-1). Among all the parasites identified in different land gradients, only *Capillaria* spp. has significantly higher prevalence in cropland area.

**Table-1:** Prevalence of gastrointestinal helminths in Suncus murinus from the southeast part of Bangladesh (n=86) (Chi-square test).

Phylum	Genus	Prevalence % (n)					p value*

		Overall prevalence (n=86)	Forest (n=22)	Dwelling (n=39)	Cropland (n=25)		
Cestode	Hymenolepis sp.	59 (51)	59 (13)	67 (26)	48 (12)	0.268	
	Taenia spp.	3 (3)	0	5 (2)	4 (1)	0.561	
Nematode	Capillaria spp.	10 (9)	4 (1)	3 (1)	28 (7)	0.003	
	Strongyloides spp.	17 (15)	23 (5)	15 (6)	16 (4)	0.768	
	Physaloptera spp.	3 (3)	0	2 (1)	8 (2)	0.317	
Trematode	Echinostoma spp.	3 (3)	5 (1)	5 (2)	0	0.518	

* p value significant at<0.05

The mean worm loads of parasitic species varied from four to six and cropland had higher worm load and showed no significant relationship with another gradient area. The relative abundance of worm in dwelling area and species diversity in cropland area was higher in comparison to other areas (Table-2).

**Table-2:** Mean worm load, relative abundance, and species diversity of helminths in Asian house shrews.

Parasites (n)	Forest (n=22)					Dwelling (n=39)					Cropland (n=25)				
		
	NSI	NPF	MWL	RA	SD	NSI	NPF	MWL	RA	SD	NSI	NPF	MWL	RA	SD
Hymenolepis spp. (51)	13	45	3.46	0.5	0.32	26	47	2	0.55	0.34	12	38	2.7	0.44	0.19
Taenia spp. (3)	0	0	0	0	0	2	100	1	1.16	0.07	1	100	1	1.16	0.03
Capillaria spp. (9)	1	8	8	0.2	0.06	1	8	12	0.09	0.04	7	8	12.1	0.09	0.14
Strongyloides spp. (15)	5	23	4.6	0.3	0.19	6	19	5.3	0.22	0.16	4	11	9.25	0.13	0.09
Physaloptera spp. (3)	0	0	0	0	0	1	25	4	0.29	0.04	2	25	4	0.13	0.05
Echinostoma spp. (3)	1	100	100	0	0.06	2	29	3.5	0.34	0.07	0	0	0	0	0
Total	20	77	3.85	0.9	0.63	36	228	4	2.65	0.72	26	182	6	2.11	0.51

NB: NSI= No. of shrew infected; NPF= No. of parasites found; MWL= Mean worm load; RA= Relative abundance; SD= Species diversity

Among the infected shrews, one or more species of parasites in a single individual were commonly found. Parasitic infestation by single species was predominant in positive shrews of all the areas. Although maximum double and multiple parasitic infestations were found in dwelling area, all areas showed double and/or multiple parasitic infestations (Table-3).

**Table-3:** Parasitic loads in individuals at different sites of the study area.

Location	Single infestation	Double infestation (%)	Multiple infestation (>2) (%)	Negative infestation (%)	Total positive (%)
Forest area (n=22)	14 (63.64)	3 (13.64)	0	5 (22.73)	17 (77.27)
Cropland (n=25)	15 (60.00)	4 (16.00)	1 (4.00)	5 (20.00)	20 (80.00)
Dwelling (n=39)	21 (53.85)	7 (17.95)	2 (5.13)	9 (23.08)	30 (76.92)

Only trematode infestation was not found in any shrew. All the trematode infestation were found in combination with cestodes and/or nematodes. Among the co-infestations, cestodes and nematodes were most common followed by cestodes and trematodes; nematodes and trematodes. Cestodes mostly infect individually in all types of land gradient.

The shrews in dwelling area were around 2 times more likely to be positive for *Hymenolepis* spp. than that of cropland. The shrews of cropland area were harboring 13 and 3 times more *Capillaria* spp.and *Physaloptera* spp., respectively, compared to shrews of dwelling area. The forest land shrews were at 2 times more risk of having *Capillaria* spp.and *Strongyloides* spp. than that of dwelling area (Table-4).

**Table-4:** Measures of strengths for different helminths corresponding to land gradients.

Parasites (n)	OR	p value*
Hymenolepididae gen. spp. (51)		
Cropland	1	
Dwelling	1.9	0.254
Forest	1.6	0.498
Taeniidae gen spp. (3)		
Cropland	1	
Dwelling	1.2	0.89
Forest	0.02	0.994
Capillaria spp. (9)		
Dwelling	1	
Cropland	12.8	0.023
Forest	2.06	0.618
Strongyloides spp. (15)		
Dwelling	1	
Cropland	1.1	0.89
Forest	2.13	0.29
Physaloptera spp.(3)		
Dwelling	1	
Cropland	3.3	0.34
Forest	0.02	0.988
Echinostoma spp. (3)		
Forest	1	
Dwelling	1.3	0.825
Cropland	0.04	0.994

* p value significant at <0.05

## Discussion

The current study explored gastro-intestinal helminthic infestation of Asian house shrews from different geographies such as dwelling, cropland, and forest areas of Bangladesh. These are the first data and the only study to consider the whole helminth spectrum of the Asian house shrews from Bangladesh. Shrews were highly infected with various GI parasites in the study. We recovered six genera of helminths which are known to have pathogenic species affecting human and animals [19,31,32]. The species diversity, and mean worm load in cropland and relative abundance in dwelling area were higher where domestic animals and human activity were predominant. The presence or abundance of parasites was somewhat modified by the geography of the study areas but only to a limited extent. Dussex *et al*. [33] have found that shrews continuously migrate from agricultural land to the nearest household resulting in the nearly similar prevalence of parasites in Crocidura shrews of different region. In another study, similar findings were reported in topographically identical countries such as Taiwan and Cambodia, where the prevalence of helminthic infestation in Asian house shrews was 100% [23] and 66.67% [4], respectively. The abundance of parasites in shrews might be mainly due to their scavenging nature. Other than the narrow home range, high population density and scavenging near the domestic animals and human population might also have potential contributions [34].

The findings of identified cestode and nematode species in the current study agree with previous reports on Asian house shrew and some species can infect humans also [19,23]. The prevalence and intensity values were somewhat higher in the previous studies [19,26] than that of the present study. The above discrepancies are difficult to explain, but the prevalence and intensity values were quite similar to those obtained by Veciana *et al*. [4].

*Taenia* spp. was found in shrews of forest and cropland areas, which is considered to be the important species to cause the most neglected tropical disease of human worldwide [35-37]. Open defecation, the intermediate host having free access to human feces, and uncontrolled slaughtering of the infected host might be linked to human–*Taenia* spp. association [38].

*Strongyloides* spp. (the pinworm or roundworm) and *Capillaria* spp.were foundto be prevalent in shrews of the cropland and dwelling areas, which was also reported in shrews in Taiwan [19]. The mean worm load, relative abundance, and species diversity of *Strongyloides* spp. and *Capillaria* spp.were higher in cropland area where human-animal activity is regular. Small mammals are being crowded near the cropland due to habitat loss, which increases the level of contamination of the environment. Thus, the worm load, relative abundance, and species diversity are increasing in the environment. Different species of *Capillaria* have been identified from *S. murinus* in Cambodia [4]. However, the present study found that 9.30% of house shrews were infected, indicating the environments might be highly contaminated with the eggs of *Capillaria* spp.Among different risk factors associated with different species of parasitic infestation, significantly greater numbers of *Strongyloides* spp.was found in adult *S. murinus*. It can be readily explained by the fact that adults have a longer period over which they accumulate the parasite. The low intensity of infestation in the juveniles makes it unlikely that *Strongyloides* spp.infestations could cause their mortality in the autumn. The other differences in nematode fauna are not so readily explained but might be a combination of the longer period over which the adults have been able to accumulate infective stages and differences in feeding habits between adult and juvenile shrews.

*Hymenolepis* spp. was the most common cestode detected in this study similar to Central Taiwan [19] and also in rodents from Southeast Asia [12,26,39,40]. Due to the conditions in which our samples were preserved (70% ethanol), we were unable to determine which species of *Hymenolepis* was recovered.

We found that shrew harbors more helminth species and has multiple species per host individual in three-land gradient. A remarkable number of researchers around the world also noticed that shrews can harbor a wide range of helminths [41]. High parasitism of the shrew may imply higher absolute food requirement that might increase the parasitic colonization rate [42], but another study suggests that high infestation levels are either due to its generalized diet or high abundance, rather than its large body size [41]. The mean worm load or intensity and prevalence are correlated with contamination ofenvironment specially soil where the parasites need to develop to become infective [32]. Presence of parasites in shrews that are also prevalent in human and domestic animals is a clear indication of invasive species. These parasites might break their species barrier and are transmitted from animals to humans or *vice versa*. Sometimes, the water sources are shared by human, domestic animals, and wildlife that might influence the exposure to infective stages of various parasites of human and domestic animal [43,44], and the intensity of infestation can change with accessibility to water resources [45]. The host health status and nutrition also play a major role in parasitic infestation load [31] by directly influencing immune responses and acquisition of immunity against the parasites [46]. Although our results demonstrated an effect of habitat on helminth species richness, it also indicated that the observed patterns may be confounded by several aspects of the biology of shrews, such as their density or ranging patterns as both of these traits have been stated as potential robust determinants of helminth diversity [47].

The genus *Hymenolepis* is most notorious, causing a pathologic effect on human health. Two genera of Platyhelminthes – *Hymenolepis* and *Echinostoma* – have been identified in shrews of Bangladesh, which was supported by a previous study as the possible origin of zoonotic helminthiasis [48]. Commensal rodents and shrews, that are associated with humans, can also play a role in continuing the life cycle of echinostomes in human settlements. Before our study, an echinostomatid in *S. murinus* was recorded in Cambodia [4]. Although *Artyfechinostomum malayanum* (a species of *Echinostome*) has been reported previously in the Asian house shrew, eggs similar with *Echinostome paraensei* from Bangladesh have also been found [49]. The higher levels of infestation of *Echinostoma* spp.in adults are likely to be due to the longer time of exposure to infective stages.

In the present study, three *S. murinus* (3.49%) were found to be infected with *Physaloptera* spp. This is the first recorded finding of stomach worm, i.e. *Physaloptera* spp. in shrews of Bangladesh. The intermediate host of *Physaloptera* spp. is cockroach, and *S. murinus* might be infected by feeding of a cockroach.

## Conclusion

These are the first data on the helminths of *S. murinus* from Bangladesh. Shrews from southeastern parts of Bangladesh had high prevalence rate and susceptibility to parasitic infestation. Two cestodes, three nematodes, and one trematode were identified in these wild shrews. Two of these parasites are of zoonotic significance, indicating the necessary step is crucial in the prevention of the transmission of zoonotic parasites. This study may also contribute to generate knowledge on the understanding of range and helminthic parasitism in house shrew from diverse regions and climatic zones of the world. The important position occupied by these animals in biocoenosis, their distribution, population density, the fact that this species cohabitate with humans, and the limited knowledge of their GI parasites in Bangladesh imply the necessity of further investigation. Studies focused on determining the prevalence of helminths infestation, risk factors for the parasitism, and the geographic distribution of helminths in shrews are needed to understand the helminth burden and guide effective prevention and control measures. Therefore, polymerase chain reaction-based molecular diagnosis and advanced scientific investigations are imperative to understand the helminth diversity and discovery of new helminth species transmission cycles and zoonotic potentials.

## Authors’ Contributions

AI and MAH designed and supervised the study; MR, SI, and MNI collected the data and conducted the study. MAH and MM assisted the laboratory experimentation and identification of parasites. MR, SI, JF, and AI were involved in data analysis and interpretation and prepared the draft of the manuscript; MA, MNUC, MMH, MAH, and AI were involved in critical revision of the manuscript. All authors discussed the results, contributed, and approved the final manuscript.
